# Indoor Localization Algorithm Based on Information Gain Ratio and Affinity Propagation Clustering

**DOI:** 10.3390/s26020664

**Published:** 2026-01-19

**Authors:** Rencheng Jin, Di Zhang, Xiao Tian, Jianping Ma

**Affiliations:** Key Laboratory for Micro/Nano Technology and System of Liaoning Province, Dalian University of Technology, Dalian 116024, China; zd_dlut@mail.dlut.edu.cn (D.Z.); xiaot@mail.dlut.edu.cn (X.T.); majianping6890@mail.dlut.edu.cn (J.M.)

**Keywords:** indoor positioning, redundant AP elimination, affinity propagation clustering, location resolution

## Abstract

In indoor positioning systems, it is common to use existing AP deployments within buildings to build a fingerprint database, providing positioning information during the online phase. However, AP layouts inside buildings often contain a large number of redundant APs, which leads to the improvement in positioning accuracy leveling off as the number of redundant APs increases, while also increasing the computational load of indoor positioning services. To address this problem, the thesis proposes a method for calculating the AP location discrimination capability and combines the location discrimination capability with coverage to eliminate redundant APs. Experiments conducted in real indoor scenarios, as well as on the Crowdsourced dataset and the SODIndoorLoc dataset, validate the results. The results show that the redundant AP removing strategy ensures that the average positioning accuracy fluctuates by no more than 5% compared to the unfiltered case, while significantly reducing the number of APs in the fingerprint database—by 64.43%, 72.78%, and 59.62%, respectively. In the position estimation phase, this paper uses affinity propagation clustering for coarse positioning and combines Bayesian methods for fine positioning. Compared with GMM, K-Means, and the pointwise algorithm, the average positioning error of the proposed method is reduced by 11% to 39%.

## 1. Introduction

Global Positioning System (GPS) signals are often severely obstructed and reflected by the structural walls of indoor buildings, leading to significant signal attenuation or even unstable reception, making it difficult to provide reliable location services [1]. As a result, indoor localization technologies have emerged as a prominent area of research. Wi-Fi, with its broad coverage, established infrastructure, and low additional hardware modification costs, offers distinct advantages [2]. The existing access points (APs) in indoor environments can directly serve as positioning infrastructure, which is why Wi-Fi signals are widely adopted in practical applications.

Wi-Fi indoor localization technology consists of two major classifications: the log-distance model (LDPLM) indoor localization technology and fingerprint-based indoor localization technology. LDPLM indoor localization technology estimates the line-of-sight (LoS) propagation distance from the Target Positions (TPs) to APs based on the LDPLM. In non-line-of-sight propagation paths, there are significant multipath effects and shadow effects, causing discrepancies between the calculated and actual propagation distances, which weaken the stability of the propagation model and reduce positioning accuracy [3]. Fingerprinting is currently the most generally employed method in Wi-Fi indoor localization [4]. Fingerprinting comprises two stages: offline and online. Researchers gather Wi-Fi RSSIs and other signal features at the reference points (RPs) along with their actual coordinates during the offline stage. The data undergoes preprocessing, including filtering, feature extraction, clustering analysis, and the removal of redundant APs, resulting in the construction of a fingerprint database. During the online stage, researchers compare instantaneous RSSI features with the fingerprint database to infer the true position of TPs [5]. Unlike methods that rely on idealized propagation models, fingerprinting establishes an empirical relationship between “signal features and spatial locations,” making it more robust in complex indoor environments characterized by non-line-of-sight propagation and shadowing effects [6].

## 2. Related Work

At the 19th IEEE Conference on Computer Communications (INFOCOM), Bahl et al., from Microsoft Research [7], first proposed the RADAR system. The system constructs a radio map inside the building based on reference points (RPs), and, during the online stage, indoor localization is achieved by matching the instantaneous RSSI vector with fingerprints in the fingerprint database.

Mainstream research efforts aimed at improving the quality of fingerprint databases, localization accuracy, and system stability encompass various strategies, including the optimization of fingerprint data, refinement of fingerprint features, and enhancement of the efficiency in fingerprint database construction. In terms of optimizing fingerprint data and feature processing, Fang et al. [8] proposed utilizing Principal Component Analysis (PCA) to reduce the scale of RSSI data, replacing the original high-dimensional data with a few principal components. Lee and Han [9] employed nonlinear autoencoder technology to reduce the dimensionality of fingerprint map data, extracting more discriminative low-dimensional features. Luo and Fu [10] proposed using Kernel Principal Component Analysis (KPCA) for nonlinear mapping of RSSI features to eliminate data redundancy in the fingerprint database while retaining useful location information.

In terms of AP selection and improving the efficiency of fingerprint database construction, Cheng et al. [11] used Support Vector Regression (SVR) to simulate unsampled or missing fingerprint data, enhancing the system’s noise resistance to environmental changes such as occlusions or human traffic. Youssef et al. [12] proposed the MaxMean method, selecting several APs with the highest RSSI at the target location to form a cluster classification mechanism based on AP selection. Pan et al. [13] combined Building Information Modeling (BIM) with a multi-layer, multi-wall signal propagation model to generate virtual fingerprints, constructing an offline fingerprint database and achieving reliable positioning accuracy. Li et al. [14] mapped Channel State Information (CSI) to a two-dimensional amplitude feature map and used amplitude-feature deep convolutional generative adversarial networks to extend the fingerprint database, significantly reducing the burden of manual data collection. Reyes et al. [15] proposed an innovative deep neural model to identify long-term time-varying data. The proposed model uses only a small amount of fingerprint information instead of the complete fingerprint database. Xiao et al. [16] combined a region-based IPS with a noise map to track the movement trajectory of objects.

These methods minimize the overheads associated with fingerprint database construction and improve feature discrimination capability; however, they do not resolve the issue of varying location resolutions across different APs. The paper proposes an algorithm for eliminating redundant APs based on location resolution evaluation, which converts the original signal strength information into a grid $Grid_{r\times c}$ and calculates the information gain ratio of each signal access point (AP). The algorithm uses information gain ratio and location coverage as ability indicators to evaluate the geographical location resolution of APs, significantly reducing the quantity of APs stored in the fingerprint dataset while ensuring positioning accuracy. In the location evaluation phase, combining affinity propagation clustering and the Bayesian algorithm, the positioning accuracy improves to varying degrees compared to KNN, the group matching method (GMM) [17], K-Means, pointwise [18], and other algorithms.

## 3. Offline Phase

### 3.1. AP Optimization via Location Resolution

Excessive and varying-quality AP information in complex indoor positioning buildings introduces signal noise, interferes with the matching process, and reduces positioning stability. Additionally, high-dimensional fingerprint databases significantly increase the cost of storage and retrieval [19]. Thus, eliminating redundant APs to create a compact and high-quality fingerprint database is crucial for improving the accuracy and maintainability of indoor Wi-Fi localization systems. Based on these considerations, this paper proposes an algorithm for eliminating redundant APs based on a location resolution assessment. The algorithm treats the signals from each access point (AP) as features, using the information gain rate as the AP location resolution capability indicator to eliminate redundant APs with lower location resolution capabilities.

#### 3.1.1. Definition of Location Resolution Ability

Assume that in a certain indoor positioning deployment, the amount of APs installed is n, and the detected signal strength is categorized into P clusters: $C_{1},C_{2},\cdots ,C_{P}$. The total number of RPs is m, and these RPs are clustered into V clusters: $U_{1},U_{2},\cdots ,U_{V}$ utilizing the K-Means algorithm based on their geographical locations. The K-Means algorithm groups geographically close RPs into the same cluster $U_{i}$. Within the same cluster, the difference between each RP is minimized, while the geographical differences between different clusters $U_{i}$ and $U_{j}$ are maximized.

The fingerprint data collected from each AP feature measurement at all reference measurement points (RPs) can be transformed into a 2D grid of size row × col. The information gain ratio for each signal access point (AP) is then calculated. The calculation method is as follows:

1. Compute the Shannon entropy of the location area, denoted as $Entropy\left(U\right)$.
  (1) $$Entropy\left(U\right)=-\sum_{v=1}^{V}\frac{\left|U_{v}\right|}{\left|U\right|}\log_{2}\frac{\left|U_{v}\right|}{\left|U\right|}$$
  where $\left|U_{v}\right|$ represents the number of RPs in cluster $U_{v}$ and $\left|U\right|$ represents the total number of RPs.
2. Calculate the conditional entropy $ConditionalEntropy\left(C|U\right)$ of each access point (AP) as a feature.
  (2) $$\begin{array}{cc} ConditionalEntropy\left(C|U\right) & =\sum_{v=1}^{V}\frac{\left|U_{v}\right|}{\left|U\right|}\left(-\sum_{p=1}^{P}\frac{\left|D_{v,p}\right|}{\left|U_{v}\right|}\log_{2}\frac{\left|D_{v,p}\right|}{\left|U_{v}\right|}\right)\\ & =-\sum_{v=1}^{V}\sum_{p=1}^{P}\frac{\left|D_{v,p}\right|}{\left|U\right|}\log_{2}\frac{\left|D_{v,p}\right|}{\left|U_{v}\right|} \end{array}$$
  where $D_{v,p}$ represents the union of $C_{p}$ and $U_{v}$, and $\left|D_{v,p}\right|$ represents the number of signal reference points (RPs) in $D_{v,p}$.
3. Calculate the information gain $InfoGain\left(C,U\right)$ of the AP as a feature.
  (3) $$\begin{array}{cc} InfoGain\left(C,U\right) & =Entropy\left(U\right)-ConditionalEntropy\left(C|U\right)\\ & =-\sum_{v=1}^{V}\frac{\left|U_{v}\right|}{\left|U\right|}\log_{2}\frac{\left|U_{v}\right|}{\left|U\right|}+\sum_{v=1}^{V}\sum_{p=1}^{P}\frac{\left|D_{v,p}\right|}{\left|U\right|}\log_{2}\frac{\left|D_{v,p}\right|}{\left|U_{v}\right|} \end{array}$$
4. Calculate the information gain ratio $InfoGainRatio\left(C,U\right)$ of each AP as a feature.
  (4) $$\begin{array}{cc} InfoGainRatio\left(C,U\right) & =\frac{InfoGain\left(C,U\right)}{SplitInfo\left(C,U\right)}\\ & =\frac{-\sum_{v=1}^{V}\frac{\left|U_{v}\right|}{\left|U\right|}\log_{2}\frac{\left|U_{v}\right|}{\left|U\right|}+\sum_{v=1}^{V}\sum_{p=1}^{P}\frac{\left|D_{v,p}\right|}{\left|U\right|}\log_{2}\frac{\left|D_{v,p}\right|}{\left|U_{v}\right|}}{-\sum_{p=1}^{P}\frac{\left|C_{p}\right|}{\left|C\right|}\log_{2}\frac{\left|C_{p}\right|}{\left|C\right|}} \end{array}$$
  where $C_{p}$ represents the set of signal strength categories for a given AP as a feature, $\left|C_{p}\right|$ represents the number of location RPs in the set $C_{p}$, and $SplitInfo\left(C,U\right)$ represents the split information of the signal strength classification.

The calculation formula for split information $SplitInfo\left(C,U\right)$ is given by Equation (5):

(5) $$SplitInfo\left(C,U\right)=-\sum_{p=1}^{P}\frac{\left|C_{p}\right|}{\left|C\right|}\log_{2}\frac{\left|C_{p}\right|}{\left|C\right|}$$

#### 3.1.2. Removing Redundant APs

In Section 3.1.1, the location resolution capabilities of each AP are first calculated and then ranked in descending order. The redundant APs, whose location resolution is in the bottom λ% and whose RP coverage is in the bottom γ%, are filtered out. The remaining APs are then used in the subsequent location estimation phase to calculate the actual position of TPs, thereby improving the overall performance and efficiency of the indoor positioning system.

### 3.2. AP Optimization via Correlation Clustering

**Figure 1:**
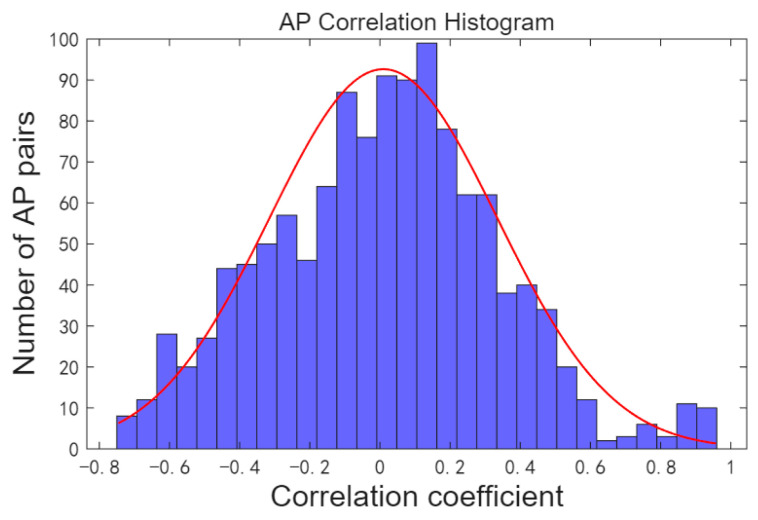
AP correlation histogram.

In the indoor positioning environment, numerous access points (APs) are deployed. For instance, the SODIndoorLoc dataset includes approximately 552 APs, with signal strength data collected for Spearman correlation analysis. The histogram of correlation statistics for each AP is presented in Figure 1, revealing that certain APs exhibit strong correlations. The higher the similarity between two APs, the stronger their interchangeability. Grouping APs with high similarity for AP optimization can reduce the feature dimension of the fingerprint database.

#### 3.2.1. Region Partitioning Based on K-Means

**Figure 2:**
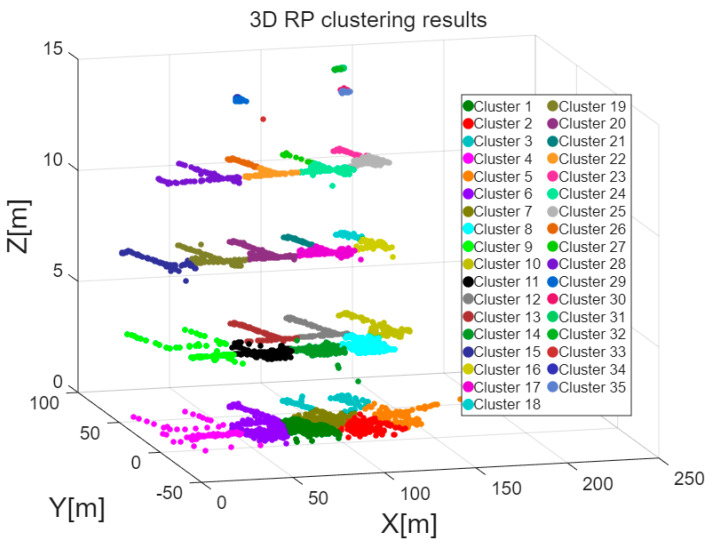
Three-dimensional RP clustering results.

**Figure 3:**
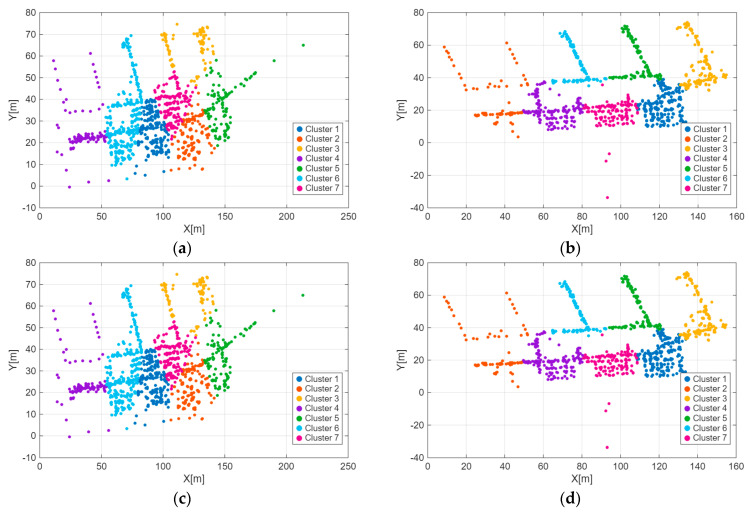
Clustering results of RPs on different floors. (**a**) Clustering results of RPs on the 1st floor. (**b**) Clustering results of RPs on the 2nd floor. (**c**) Clustering results of RPs on the 3rd floor. (**d**) Clustering results of RPs on the 4th floor.

As outlined in Section 3.1.1, calculating the information gain as an indicator of AP location resolution requires dividing the area into distinct location zones and forming a two-dimensional grid. The K-Means algorithm groups location reference points that are geographically close to the same cluster. In this study, the publicly available dataset published by Lohan et al. [20] is used to demonstrate the K-Means clustering results. The dataset was collected from a four-floor university building in Tampere, Finland, between January and August 2017, and includes 687 training fingerprints and 3951 test fingerprints. Figure 2 shows the results of location reference point clustering using the K-Means method for the entire location estimation dataset, while Figure 3 displays the regional division for each floor (first to fourth) of the building. As illustrated in Figure 2 and Figure 3, the K-Means algorithm effectively clusters the location reference points (RPs) in the dataset based on their geographical locations, which is then applied in Section 3.2.3 for the calculation of location resolution.

#### 3.2.2. AP Correlation Clustering

Assume that the signal strength vector $V_{p}=\left(rssi_{1}^{p},rssi_{2}^{p},rssi_{3}^{p},\ldots ,rssi_{m}^{p}\right)$ of the signal access point $AP_{p}$ consists of the signal strength measurements at the RP, and the signal strength vector $V_{q}=\left(rssi_{1}^{q},rssi_{2}^{q},rssi_{3}^{q},\ldots ,rssi_{m}^{q}\right)$ of the signal access point $AP_{q}$ consists of the signal strength measurements at each RP. After normalizing the signal vectors $V_{p}$ and $V_{q}$, the maximum mutual information between $AP_{p}$ and $AP_{q}$ is calculated to obtain their similarity. The calculation steps are as follows:

1. Collecting Wi-Fi Fingerprint Signals

For any two signal access points, $AP_{p}$ and $AP_{q}$, a signal sample set $D=\left\{\left(rssi_{p}^{i},rssi_{q}^{j}\right)|i=1,2,\ldots ,N\right\}$ is constructed, $D=\left\{\left(rssi_{p}^{i},rssi_{q}^{j}\right)|i=1,2,\ldots ,N\right\}$, $V_{p}=\left(rssi_{1}^{p},rssi_{2}^{p},rssi_{3}^{p},\ldots ,rssi_{m}^{p}\right)$, and $V_{q}=\left(rssi_{1}^{q},rssi_{2}^{q},rssi_{3}^{q},\ldots ,rssi_{m}^{q}\right)$, where N denotes the quantity of samples.

2. Computation of Maximum Mutual Information

The signals of $V_{p}$ and $V_{q}$ are converted into two-dimensional scatter plots, which are then divided into r × c smaller regions, satisfying the constraint Formula (6).

(6) $$r\times c<B\left(N\right)$$

where $B\left(N\right)$ represents the threshold for the number of region divisions, typically set as $B\left(N\right)=N^{0.6}$ [21] and N represents the total data volume.

Calculate the mutual information $I\left(AP_{p};AP_{q}|G\right)$ for each region division scheme. Different partitioning schemes exist for the same grid size. Mutual information is normalized based on the grid partitioning scale, and the maximum mutual information value is selected as the MIC. The calculation formula is as follows:

(7) $$I\left(AP_{p};AP_{q}\right)=\underset{r\times c<B\left(N\right)}{\max}\left\{\frac{I{\left(AP_{p};AP_{q}|G\right)}_{r,c}}{\log_{2}\min\left\{r,c\right\}}\right\}$$

where $I{\left(AP_{p};AP_{q}|G\right)}_{r,c}$ represents normalized mutual information values for different grid partitioning scales.

3. Signal Access Point Clustering

The maximum mutual coefficient for all pairs of signal access points Ap is used to form a fuzzy relation matrix M. The fuzzy equivalence matrix M* and its λ-cut matrix $M_{\lambda }^{*}$ are then computed. $M_{\lambda }^{*}$ satisfies Equations (8) and (9).

(8) $$M_{\lambda }^{*}=\left[\begin{array}{cccc} {\tilde{m}}_{11}^{*} & {\tilde{m}}_{12}^{*} & \cdots & {\tilde{m}}_{1n}^{*}\\ {\tilde{m}}_{21}^{*} & {\tilde{m}}_{22}^{*} & \cdots & {\tilde{m}}_{2n}^{*}\\ \vdots & \vdots & \ddots & \vdots \\ {\tilde{m}}_{n1}^{*} & {\tilde{m}}_{n2}^{*} & \cdots & {\tilde{m}}_{nn}^{*} \end{array}\right]$$

(9) $${\tilde{m}}_{ij}^{*}=\left\{\begin{array}{c} 1,{\tilde{m}}_{11}\geq \beta \\ 0,{\tilde{m}}_{11}<\beta \end{array}\right.$$

Clustering of signal access points (APs) is performed based on the λ-cut matrix. The signal access points are assigned to groups $S_{1},S_{2},\cdots ,S_{K}$.

#### 3.2.3. Removing Redundant APs

In the same clustering group $S_{k}$, the signal access points (APs) exhibit high similarity and can be interchangeable. Therefore, a signal access point with higher position resolution is selected within $S_{k}$ to construct the fingerprint features. In this section, the position resolution metric from Section 3.1.1 is also used as the evaluation criterion for resolution among APs within the same cluster. The AP with the highest position resolution is selected from any similar AP cluster $S_{k}$ to represent $S_{k}$ and participate in constructing the fingerprint database.

## 4. Online Phase

Common clustering methods used in indoor positioning systems include K-Means clustering [22,23], variants of K-Means clustering [24,25,26], and fuzzy clustering [27]. The affinity propagation clustering method, proposed by Frey and Dueck [28], distinguishes itself from other clustering methods by not requiring the pre-selection of initial cluster points or the number of clusters. In this paper, during the position evaluation phase, affinity propagation clustering is applied to cluster the location reference points (RPs), forming clusters $S_{1},S_{2},\cdots ,S_{K}$, which complete the coarse positioning. The top N most similar clusters, $S_{k},S_{k+1},\cdots ,S_{k+n}$, are selected as the clusters used in the fine positioning phase. Gaussian probability estimation is performed on the fingerprint dataset contained within the clusters, and Bayesian position estimation is applied based on the Gaussian probabilities to determine the final location of the point to be located. The computational complexity of the core algorithm for position prediction is O(M × (C + N)), where M represents the number of test samples, N represents the number of training samples, and C represents the number of clusters. To present this process more clearly, Algorithm 1 shows the pseudocode for the key parts of the position estimation phase, which facilitates a better understanding of the implementation details of the method proposed in this paper.
**Algorithm 1** Indoor Localization PredictionInput: Training fingerprints D = {(r_i_, y_i_)}_{i = 1..m}, AP set A = {a_1_..a_n_}Output: Estimated position ŷ// --------------- Offline: AP discriminability evaluation and redundancy filtering -------------  1: Cluster RP coordinates {y_i_} into V clusters via KMeans → C(y_i_) // region partition  2: Compute region entropy H(C)       // entropy of location clusters  3: for each AP a in A do           // treat each AP as a feature  4:   Discretize RSSI values {r_i_[a]} into P bins → S_a_  // RSSI category set for AP a  5:   Compute conditional entropy H(C | a) using S_a_
 6:   IG(a) ← H(C) − H(C | a)         // information gain  7:   SI(a) ← SplitInfo(S_a_)          // penalty term for many bins  8:   IGR(a) ← IG(a)/SI(a)        // InfoGainRatio as discriminability  9: end for  10: Rank APs by IGR(a) in descending order    // higher = better location resolution  11: Keep top-K APs (or threshold-based) → A_sel_; discard the rest as redundant   // build compact fingerprint DB  12: Build reduced training set D_sel_ using only A_sel_  // dimension-reduced fingerprint library  // -------- Online: Position evaluation (coarse + fine localization) --------  13: Compute similarity matrix S(i,j) on D_sel_ using log-Gaussian distance   // similarity for clustering  14: Run Affinity Propagation on D_sel_ → clusters G_k_ with exemplars e_k_  // coarse clusters, no K preset  15: For test r*, compute sim(r*, e_k) for all exemplars, select top-N clusters   // coarse localization  16: Candidate RPs Ω ← union of RPs in the selected clusters       // restrict search space  17: for each RP i in Ω do  18:   p_i_ ← GaussianLikelihood(r* | r_i_, σ)                    // Bayes likelihood from RSSI gap  19:   score_i_ ← log(p_i_)                             // posterior proxy score  20: end for  21: Select top-M RPs by score_i_ → Ω_M                        // best-matching reference points  22: ŷ ← weighted_average({y_i_ | i∈Ω_M}, weights = score_i_)              // final position estimation  23: return ŷ

### 4.1. Affinity Propagation-Based Coarse Positioning

Affinity propagation clustering treats each sample data point as a potential cluster center candidate. A similarity matrix $Q_{n\times n}$ is constructed between the sample data points, where the similarity $q_{i,j}$ between each pair of data points is determined by the logarithmic Gaussian distance $L_{i,j}$. The formula for calculating $L_{i,j}$ is as follows:

(10) $$L_{i,j}=\ln\left(\frac{1}{\sqrt{2\pi }\sigma }\exp\left(-\frac{{\left(rssi_{i}-rssi_{j}\right)}^{2}}{2\sigma^{2}}\right)\right)$$

where $\sigma^{2}$ represents the shadow variance of Wi-Fi signal strength and $rssi_{i}$ represents the RSSI vector of the i-th RP.

In affinity propagation clustering, responsibility $r_{i,j}$ and availability $a_{i,j}$ are used to measure the suitability of a data point as a cluster representative for other data points. These values are iteratively updated until convergence is reached. For the i-th sample data point, the values of $r_{i,j}$ and $a_{i,j}$ are exchanged to maximize $r_{i,j}+a_{i,j}$. A sample data point j is selected as the cluster center if it satisfies the condition $r_{i,j}+a_{i,j}>0$.

### 4.2. Bayesian-Based Fine Positioning

Using affinity propagation clustering, all location reference points (RPs) are divided into K clusters, and the cluster centers of each cluster are obtained. These cluster centers are then used to assess the top n best clusters $S_{k},S_{k+1},\cdots ,S_{k+n}$ in the coarse positioning stage. Based on the logarithmic Gaussian distance and prior conditions, Bayes’ theorem is used to update the posterior distribution and estimate the localization of TPs.

In the Bayesian position estimation phase, the training set is defined as the RPs and the likelihood distribution of the RPs. The difference vector between the position of the TPs and RPs in the training set is used to calculate the Gaussian PDF. The matching probability $P\left(rssi_{TP}|loc_{RP}\right)$ of the RSSI vectors between the TPs and the RPs is then calculated, as shown in Formula (11).

(11) $$P\left(rssi_{TP}|loc_{RP}\right)=\prod_{m}\frac{1}{\sqrt{2\pi }\sigma }\exp\left(-\frac{{\left(rssi_{TP,m}-rssi_{RP,m}\right)}^{2}}{2\sigma^{2}}\right)$$

where $rssi_{TP}$ represents the RSSI vector detected at the TP, $rssi_{RP,m}$ represents the RSSI vector detected at the RP, $rssi_{TP,m}$ represents the RSSI detected at the TP from $AP_{m}$, $loc_{RP}$ represents the position vector at the location reference point (RP), and $\sigma^{2}$ represents the shadow variance of the RSSI.

The logarithm of the matching probability $P\left(rssi_{TP}|loc_{RP}\right)$ between the TP and the RP is taken as the proxy posterior score for the position of the RP. The top N RPs with the highest scores are selected as candidate RPs. The final position of the TP is calculated by performing a weighted average of the localization of the candidate RPs.

## 5. Model Estimation and Experimental Results

This section validates the superiority of the model proposed in the paper compared to other indoor positioning methods introduced in recent years. Experiments were conducted in a long corridor on the sixth floor of the Zhifang Building at Dalian University of Technology to measure positioning errors. To facilitate comparison with other positioning methods proposed in recent years, the performance of the model was also evaluated using the Tempare and SODIndoorLoc datasets.

**Figure 4:**
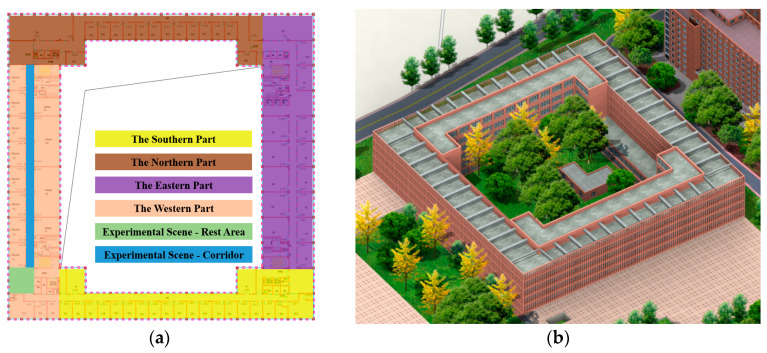
Environmental layout of the sixth floor of the Zhifang Building. (**a**) Interior layout of the Zhifang building. (**b**) Exterior shape of the Zhifang building.

**Figure 5:**
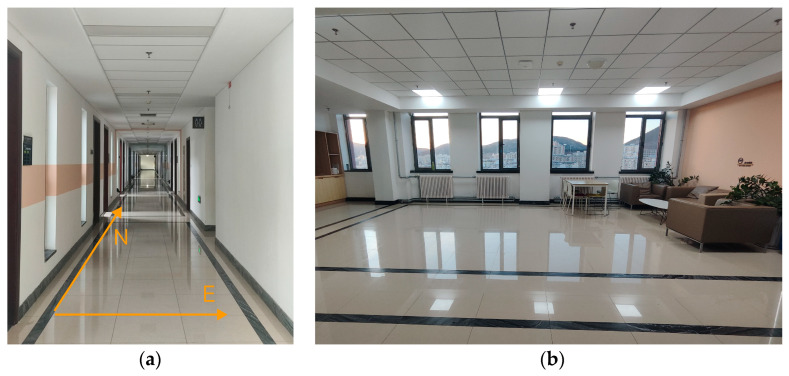
Environmental scenarios. (**a**) Long corridor experimental scene. (**b**) Rest area experimental scene.

The Zhifang Building at Dalian University of Technology, located at longitude 121.52 and latitude 38.89, has a total of nine floors, with each floor’s architectural layout being quite similar. This study conducted the positioning effect validation experiment on the sixth floor of the Zhifang Building, and a 2D floor plan of the sixth floor is shown in Figure 4. Some experimental scenes are shown in Figure 5. The spacing between each RP is approximately 0.6 m.

The Crowdsourced dataset [20] is an indoor fingerprint dataset collected via crowdsourcing from January to August 2017 within a four-story building at Tampere University of Technology in Finland. The building covers an area of approximately 22,570 square meters. The dataset consists of two subsets: the training set, which contains about 687 fingerprint samples, and the testing set, which contains about 3951 fingerprint samples. The quantity of APs is approximately 992.

The SODIndoorLoc dataset [29] is an extension of the classic UJIIndoorLoc dataset, covering three buildings: CETC331, HCXY, and SYL, with a total covered area of approximately 8000 square meters. The indoor positioning scenarios include various building environments, such as offices, corridors, and meeting rooms. The dataset contains about 23,925 fingerprint samples, with the training set consisting of approximately 21,205 samples and the testing set containing about 2720 samples. The number of signal access points is around 762. The spacing between each RP in the building numbered SYL and the building numbered HCXY is approximately 1.2 m, while the spacing between each RP in the CETC building is approximately 0.5 m.

The experimental scenario involves factors such as movement of individuals and door openings and closings, as well as obstacles like sofas, tables, chairs, electrical appliances, and walls. The fingerprint database for the experiment is collected in this environment, where algorithm validation is performed, meeting the dynamic and complex conditions required for indoor positioning. The Crowdsourced dataset constructs a fingerprint database for a multi-story building, utilizing crowdsourcing to collect fingerprint data from 21 different mobile devices and users. The data collection period for the Crowdsourced dataset spans approximately 7 months, fulfilling the temporal dynamics requirement for indoor positioning algorithm validation. The SODIndoorLoc dataset covers various indoor environments, including offices, meeting rooms, and corridors, with around 20,000 data samples. The indoor environments contain diverse obstacles, meeting the complexity requirements for indoor positioning algorithm validation.

### 5.1. Comparison of AP Optimization Effects

In Section 3, two AP optimization schemes were proposed: Scheme 1—AP Redundancy Optimization Algorithm Based on Position Resolution Evaluation, and Scheme 2—AP Optimization Algorithm Based on Correlation Grouping and Position Resolution Filtering. This section compares the positioning results of these two schemes in terms of AP reduction ratio, positioning error distribution, average positioning error, and other performance metrics.

**Figure 6:**
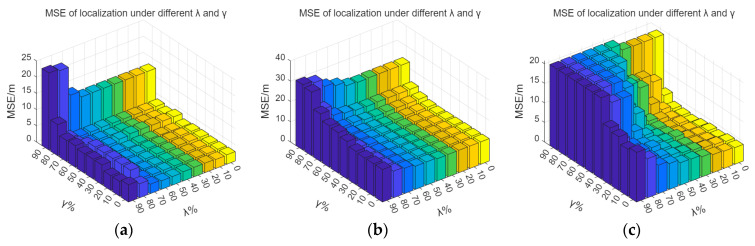
MSE of localization under different λ and γ. (**a**) Average localization error in experimental scenario under different λ and γ. (**b**) Average localization error in the Crowdsourced dataset under different λ and γ. (**c**) Average localization error in SODIndoorLoc dataset under different λ and γ.

**Figure 7:**
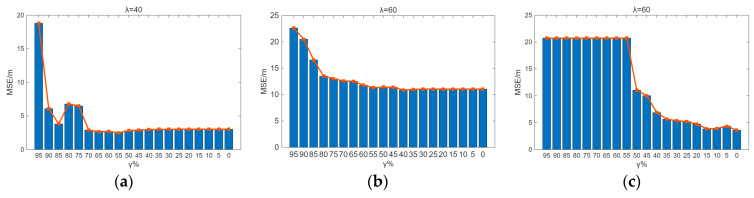
MSE of localization under different γ with fixed λ. (**a**) Average localization error in experimental scenario under different γ with fixed optimal λ. (**b**) Average localization error in Crowdsourced dataset under different γ with fixed optimal λ. (**c**) Average localization error in SODIndoorLoc dataset under different γ with fixed optimal λ.

**Figure 8:**
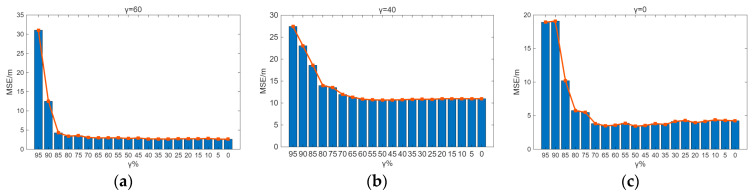
MSE of localization under different λ with fixed γ. (**a**) Average localization error in experimental scenario under different λ with fixed optimal γ. (**b**) Average localization error in Crowdsourced dataset under different λ with fixed optimal γ. (**c**) Average localization error in SODIndoorLoc dataset under different λ with fixed optimal γ.

In Scheme 1, after filtering the signal access points (APs) based on position coverage of λ% and position resolution of γ%, the MSE of indoor localization on the fingerprint database is shown in Figure 6. Figure 6, Figure 7 and Figure 8 illustrate that as λ and γ decrease, the average positioning error shows a decreasing trend, with the rate of decrease gradually slowing down. The more APs there are, the more available information the fingerprint database provides for positioning services, leading to a gradual reduction in positioning errors. However, once the quantity of APs reaches a certain threshold, additional APs result in only marginal gains in positioning accuracy.

**Figure 9:**
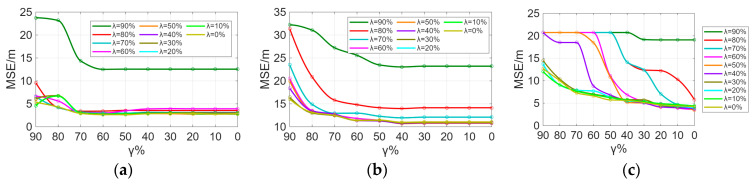
γ sensitivity analysis. (**a**) Sensitivity analysis of γ in the experimental scenario. (**b**) γ sensitivity analysis of the Crowdsourced dataset. (**c**) γ sensitivity analysis of the SODIndoorLoc dataset.

**Figure 10:**
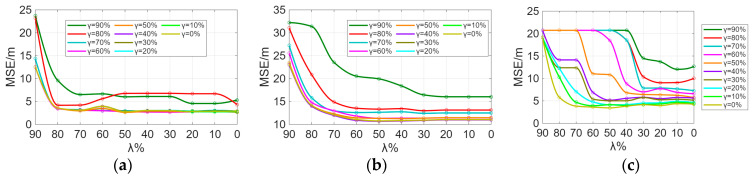
λ sensitivity analysis. (**a**) Sensitivity analysis of λ in the experimental scenario. (**b**) λ sensitivity analysis of the Crowdsourced dataset. (**c**) λ sensitivity analysis of the SODIndoorLoc dataset.

Sensitivity analysis was performed on the parameters λ and γ. From Figure 9 and Figure 10, it can be observed that the indoor positioning mean square error gradually decreases as λ and γ decrease, with the rate of decrease slowing down. In the experimental scenario, the indoor positioning mean square error remains almost unchanged at γ = 60% and λ = 50%. In the Crowdsourced dataset and SODIndoorLoc dataset, the indoor positioning mean square error remains almost unchanged at γ = 50% and λ = 30%, and γ = 30% and λ = 30%, respectively. Figure 9 and Figure 10 further validate that when the number of APs increases to a certain level, the improvement in positioning accuracy due to the increase in the number of APs becomes limited.

By traversing λ and γ, it is found that in the real indoor experimental scenario, the Crowdsourced dataset, and the SODIndoorLoc dataset, the minimum number of APs can be achieved with λ = 40, γ = 60; λ = 60, γ = 40; and λ = 60, γ = 0, respectively. Furthermore, the increase in the MSE of indoor localization compared to the original MSE of indoor localization does not exceed 5%. After the original fingerprint database undergoes AP redundancy processing and is combined with KNN for position estimation, the mean positioning errors for the experimental scenario, the Crowdsourced dataset, and the SODIndoorLoc dataset are 2.69 m, 10.88 m, and 3.60 m, respectively.

**Figure 11:**
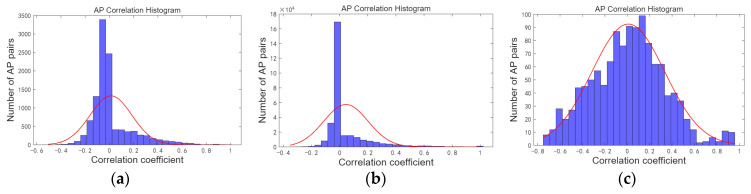
AP correlation histogram. (**a**) Correlation histogram of all AP signals in experimental scenario. (**b**) Correlation histogram of all AP signals in Tempare dataset. (**c**) Correlation histogram of all AP signals in SODIndoorLoc dataset.

Scheme 2 groups APs with high similarity into the same cluster $S_{k}$ and filters out APs with lower position resolution within $S_{k}$ to construct the fingerprint database. The Spearman correlation statistics for the APs in the three indoor positioning fingerprint datasets are shown in Figure 11. The proportion of APs with strong correlation (correlation coefficient η satisfying $\left|\eta \right|>0.8$) is relatively small.

The quantity of APs used and the MSE of indoor positioning in the positioning experiments conducted on the three datasets are illustrated in Table 1. The number of strongly correlated APs that can be excluded in Scheme 2 is far fewer than in Scheme 1. Scheme 1 can use fewer APs while maintaining similar positioning accuracy. Specifically, with Scheme 1, the number of APs used was reduced by 64.43%, 72.78%, and 59.62% for the three datasets, respectively, while Scheme 2 only reduced the number of APs by approximately 1.34%, 0.40%, and 61.54%.

**Figure 12:**
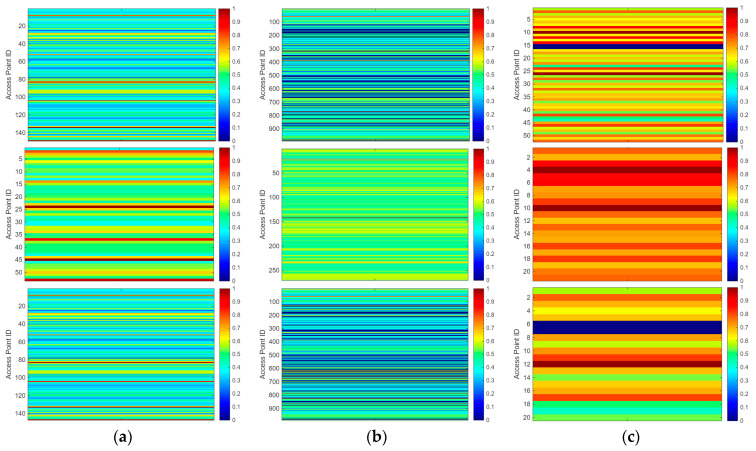
Heatmap of AP location resolution (initial, Schemes 1 and 2). (**a**) Raw fingerprint database signal heatmap. (**b**) Fingerprint database signal heatmap after processing with Scheme 1. (**c**) Fingerprint database signal heatmap after processing with Scheme 2.

The heatmaps of the AP location resolution for the initial fingerprint database, Schemes 1 and 2 are shown in Figure 12. The closer the color is to warm tones, the higher the AP location resolution. As shown in Figure 12, the fingerprint database in Scheme 1 exhibits higher AP location resolution.

**Figure 13:**
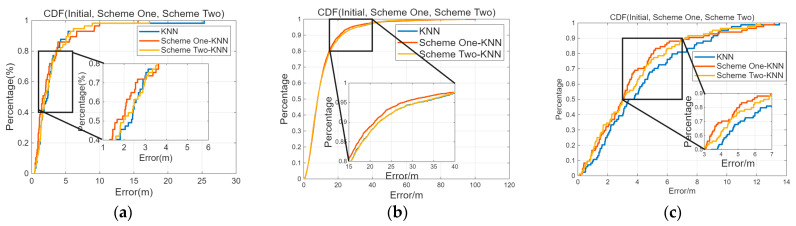
CDF (initial, Schemes 1 and 2). (**a**) Localization error CDF on the 6th floor of experimental scenario under different schemes. (**b**) Localization error CDF in the Crowdsourced dataset under different schemes. (**c**) Localization error CDF in the SODIndoorLoc dataset under different schemes.

The error CDF images for the initial fingerprint database, Schemes 1 and 2 in the three experimental scenarios are shown in Figure 13. Both Schemes 1 and 2 can still ensure positioning accuracy similar to the initial fingerprint database, despite the reduced quantity of APs, and the increase in positioning error is kept within 5% compared to before processing. In the experimental scenario, the Crowdsourced dataset, and the SODIndoorLoc dataset, Scheme 1 slightly outperforms Scheme 2 in terms of positioning accuracy.

**Figure 14:**
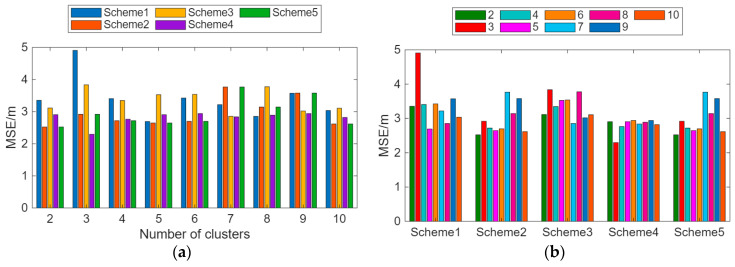
Average positioning error under different partitioning methods. (**a**) Average positioning error under different RSSI strength partitioning schemes. (**b**) Average positioning error for different numbers of clusters.

Scheme 1 uses the K-Means clustering method to divide the RPs into V clusters and the RSSI values into P clusters. This paper also investigates the impact of five different RSSI strength partitioning schemes and the number of RP clusters on positioning accuracy. Table 2 presents the five different RSSI strength partitioning schemes, and Figure 14 displays the average positioning errors under different partitioning methods. From Figure 14a, it can be observed that Schemes 4 and 5 in the RSSI partitioning schemes have relatively small positioning errors. Figure 14b indicates that in the experimental scenario, clustering numbers between Schemes 4 and 6 can achieve higher positioning accuracy.

In summary, Scheme 1 can ensure that the increase in the average indoor positioning error does not exceed 5% while reducing the number of APs used by approximately 50%, demonstrating superior performance compared to Scheme 2. Therefore, Scheme 1 is chosen as the technical approach for fingerprint database processing in this study.

### 5.2. Comparison of Indoor Positioning Errors

The indoor positioning algorithm proposed in the paper identifies the position reference point clusters to which the TP belongs during the coarse localization stage. It then selects the top n most similar RP clusters $S_{k},S_{k+1},\cdots ,S_{k+n}$ to the TP. The results of affinity propagation clustering on the experimental scenario and the SODIndoorLoc-CETC331 building dataset are shown in Figure 13. It can be seen that affinity propagation clustering effectively clusters the location reference points (RPs) based on signal strength similarity. During the fine positioning stage, the RPs in clusters $S_{k},S_{k+1},\cdots ,S_{k+n}$ are used as candidate reference points for Bayesian position estimation.

Table 3 presents the floor identification accuracy of the proposed method on two public fingerprint datasets, achieving rates of 90.00% and 95.24%, respectively, which demonstrates high performance in floor determination.

**Figure 15:**
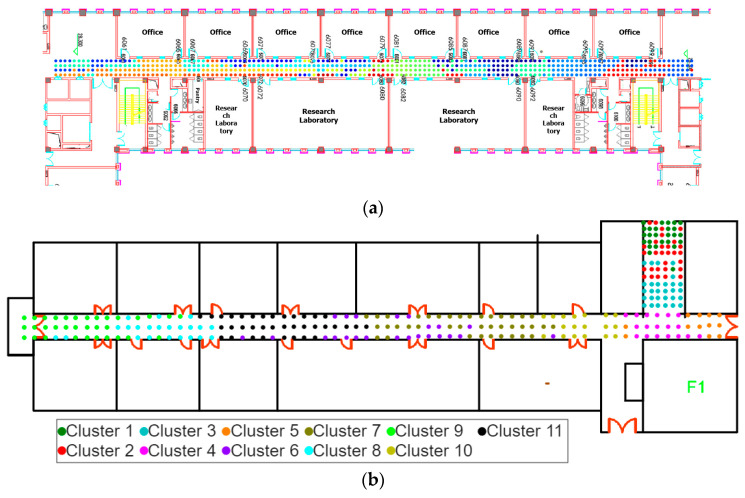

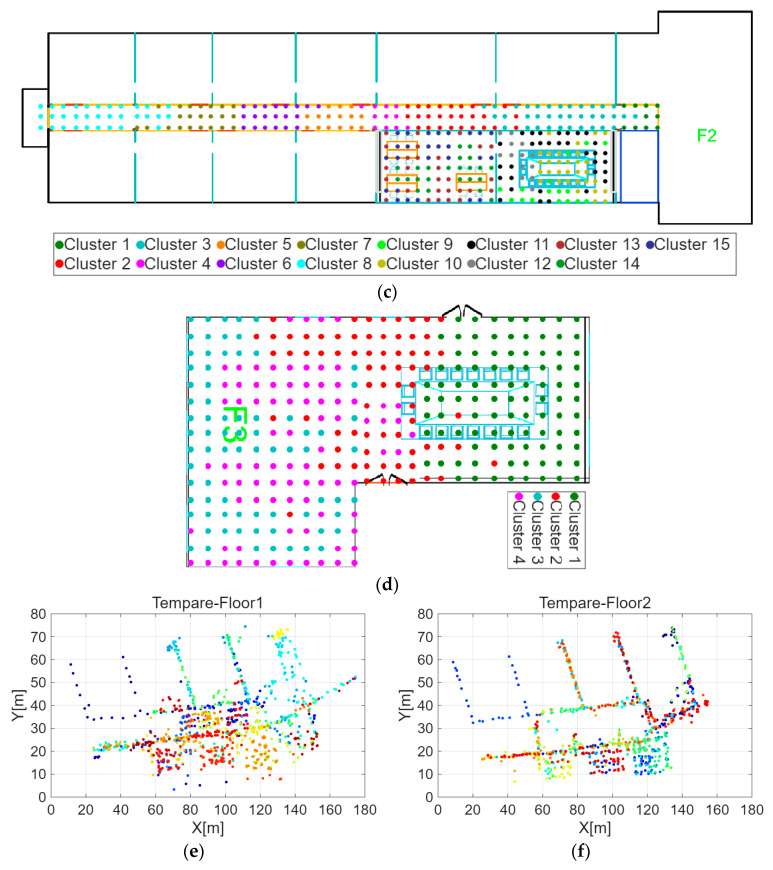
Affinity propagation clustering results. (**a**) RP clustering results on the experimental scenario. (**b**) RP clustering results on the 1st floor of CETC331 building. (**c**) RP clustering results on the 2nd floor of CETC331 building. (**d**) RP clustering results on the 3rd floor of CETC331 building. (**e**) RP clustering results on the 1st floor of Crowdsourced dataset. (**f**) RP clustering results on the 2nd floor of Crowdsourced dataset.

**Figure 16:**
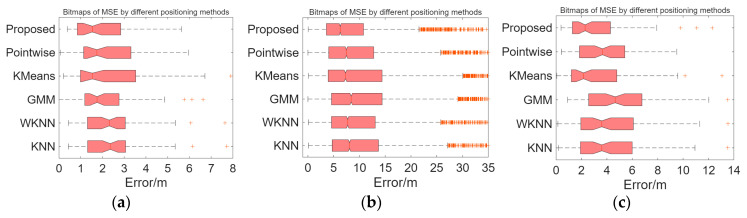
Boxplot of positioning errors for various indoor positioning methods. (**a**) Average localization error boxplot for the experimental scenario. (**b**) Average localization error boxplot for the Crowdsourced dataset. (**c**) Average localization error boxplot for the SODIndoorLoc dataset.

Table 4 presents the MSE of the algorithm proposed in the paper compared to other positioning methods. Figure 15 illustrates the distribution of positioning errors for different methods. Compared to the KNN, WKNN, GMM [17], K-Means clustering-Bayesian [30], and Pointwise Coverage Area [18] methods, the proposed method exhibits a smaller average positioning error. Figure 16 displays the CDF statistics of positioning errors for these methods on the experimental scenario, Crowdsourced dataset, and SODIndoorLoc-CETC331 building dataset. Due to differences in fingerprint collection density, the positioning error distributions vary across the datasets.

A large number of scholars [4,6,13,31,32] have studied the impact of RP collection density on indoor positioning accuracy. Shang and Wang [4] emphasized the importance of RP density for positioning accuracy, noting that an increased number of RPs allows for a more detailed signal distribution, but also increases collection costs and computational overheads. Ayub et al. [32] pointed out that high-density RP collection can improve the accuracy of indoor positioning service systems. The research on RP density by previous scholars is already quite comprehensive, so this paper does not further investigate the impact of RP collection density on indoor positioning errors.

Using the proposed model, for the experimental scenario, 80% of the positioning errors are within 3.25 m, and 95% are within 5.44 m. For the Crowdsourced dataset, 80% of the positioning errors are within 12.46 m, and 95% are within 24.45 m. For the SODIndoorLoc dataset, 80% of the positioning errors are within 4.56 m, and 95% are within 10.19 m. The Crowdsourced dataset and SODIndoorLoc dataset are both multi-story building fingerprint database datasets. The method proposed in this paper achieves floor correct identification rates of 90.00% and 95.24% for the Crowdsourced and SODIndoorLoc datasets, respectively, which basically meet the service requirements of indoor positioning systems.

**Figure 17:**
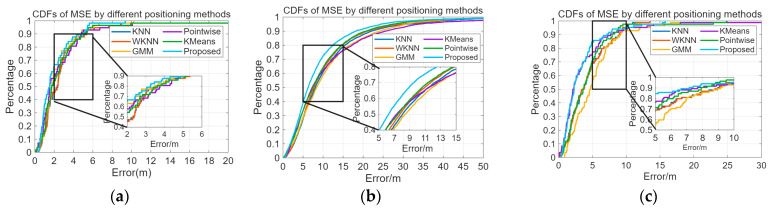
CDF statistics of positioning errors for various indoor positioning methods. (**a**) CDF of localization error for different localization methods on experimental scenario. (**b**) CDF of localization error for different localization methods in Crowdsourced dataset. (**c**) CDF of localization error for different localization methods in SODIndoorLoc dataset.

**Figure 18:**
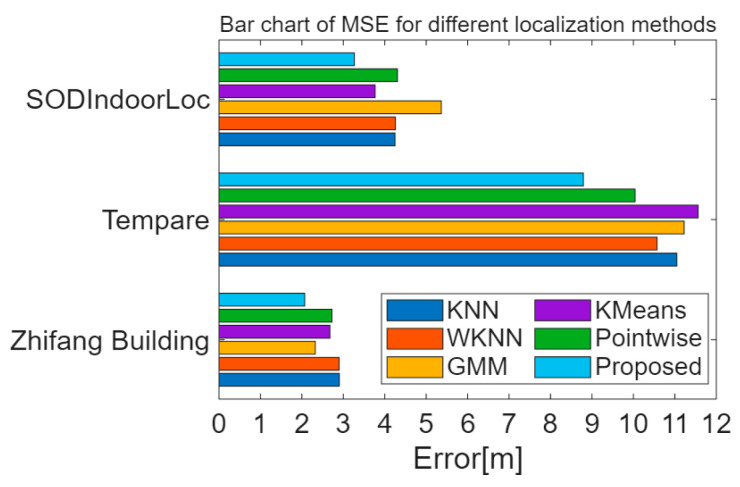
Bar chart of MSE for different localization methods.

Figure 17 and Figure 18 show the average positioning errors for different positioning models on the experimental scenario, Crowdsourced dataset, and SODIndoorLoc-CETC331 building dataset. The proposed positioning model demonstrates higher positioning accuracy. Compared to GMM positioning, the positioning errors for the experimental scenario, Crowdsourced dataset, and SODIndoorLoc-CETC331 building dataset are reduced by approximately 11.02%, 21.65%, and 39.10%, respectively. Compared to the K-Means clustering method, the positioning errors are reduced by 22.73%, 23.94%, and 13.31%, respectively. Compared to the pointwise method, the positioning errors are reduced by 24.09%, 12.41%, and 24.12%, respectively.

## 6. Conclusions

The paper proposes a Wi-Fi fingerprint indoor positioning strategy based on the information gain ratio and affinity propagation clustering. A method for calculating the position resolution of APs is defined, and redundant APs are removed based on position resolution and position coverage. The effectiveness of the proposed method was validated through experiments conducted using the Tampere dataset, the SODIndoorLoc dataset, and a real-world experimental scenario.

The experimental results demonstrate that the proposed AP redundancy removal strategy effectively decreases the quantity of APs stored in the fingerprint dataset, while preserving positioning accuracy. The number of APs is reduced by 64.43%, 72.78%, and 59.62%, respectively. In the position estimation phase, coarse positioning is performed using affinity propagation clustering, and fine positioning is achieved through Bayesian methods.

Compared to GMM positioning, the positioning errors in the experimental scenario, Crowdsourced dataset, and SODIndoorLoc-CETC331 building dataset are reduced by approximately 11.02%, 21.65%, and 39.10%, respectively. When compared to the K-Means clustering method, positioning errors decrease by 22.73%, 23.94%, and 13.31%, respectively. Compared to the pointwise method, positioning errors are reduced by 24.09%, 12.41%, and 24.12%, respectively.

## Figures and Tables

**Table 1:** MSE and amount of APs using Schemes 1 and 2.

	Methods	Scheme	Error/m	Number of APs	Initial Number of APs
Experiment	KNN	Scheme 1	2.69	53	149
		Scheme 2	2.71	147	149
	WKNN	Scheme 1	2.90	53	149
		Scheme 2	2.79	147	149
	GMM	Scheme 1	2.73	53	149
		Scheme 2	2.33	147	149
Tempare	KNN	Scheme 1	10.88	270	992
		Scheme 2	11.04	988	992
	WKNN	Scheme 1	10.45	270	992
		Scheme 2	10.58	988	992
	GMM	Scheme 1	11.16	270	992
		Scheme 2	11.22	988	992
SODIndoorLoc	KNN	Scheme 1	3.60	21	52
		Scheme 2	3.63	20	52
	WKNN	Scheme 1	3.63	21	52
		Scheme 2	3.66	20	52
	GMM	Scheme 1	5.66	21	52
		Scheme 2	6.02	20	52

**Table 2:** Different RSSI strength partitioning schemes.

Scheme	Details					
	Excellent	Good	Fair	Poor	Very Poor	No Signal
**Scheme 1**	≥−55	[−77, −55)	[−88, −77)	[−100, −88)	-	<−100
**Scheme 2**	≥−50	[−60, −50)	[−70, −60)	[−80, −70)	-	<−80
**Scheme 3**	≥−45	[−55, −45)	[−65, −55)	[−75, −65)	[−85, −75)	<−85
**Scheme 4**	-	≥−60	[−70, −60)	<−70	-	-
**Scheme 5**	≥−50	[−60, −50)	[−70, −60)	[−80, −70)	-	<−80

**Table 3:** Correct floor identification rate for different positioning methods.

	Method	KNN	WKNN	GMM	K-Means	Pointwise	Proposed
Dataset							
**Tempare**		81.12%	81.83%	82.11%	82.59%	86.64%	90.00%
**SODIndoorLoc**		98.81%	98.81%	97.62%	98.81%	97.62%	95.24%

**Table 4:** MSE of positioning for different methods. (Unit: meters.)

	KNN	WKNN	GMM	K-Means	Pointwise	Proposed
**Experiment**	2.90	2.90	2.32	2.68	2.73	2.07
**Crowdsourced**	11.05	10.57	11.22	11.56	10.04	8.79
**SODIndoorLoc**	4.25	4.26	5.36	3.77	4.30	3.27

## Data Availability

Data available on request due to restrictions. The data presented in this study are available on request from the corresponding author due to the policies and confidentiality agreements followed by our laboratory. Additionally, we used data from the publicly available Zenodo dataset at https://zenodo.org/records/1001662, reference number 10.5281/zenodo.889798, and github at https://doi.org/10.1186/s43020-022-00086-y, reference number 10.1186/s43020-022-00086-y. These data were derived from the following resources available in the public domain: Crowdsourced dataset: https://zenodo.org/records/1001662 (accessed on 8 November 2022). SODIndoorLoc dataset: https://github.com/bijingxue/SODIndoorLoc (accessed on 12 September 2017).

## Abbreviations

The following abbreviations are used in this manuscript:

APs	Access Points
RPs	Reference Points
KNN	K-Nearest-Neighbor
WKNN	Weighted K-Nearest Neighbor
CDF	Cumulative Distribution Function
RSSI	Received Signal Strength Indication
MIC	Maximum Information Coefficient
